# *Aphanizomenon flos-aquae* (AFA) Extract Prevents Neurodegeneration in the HFD Mouse Model by Modulating Astrocytes and Microglia Activation

**DOI:** 10.3390/ijms24054731

**Published:** 2023-03-01

**Authors:** Giacoma Galizzi, Irene Deidda, Antonella Amato, Pasquale Calvi, Simona Terzo, Luca Caruana, Stefano Scoglio, Flavia Mulè, Marta Di Carlo

**Affiliations:** 1 Istituto per la Ricerca e l’Innovazione Biomedica (IRIB), CNR, via U. La Malfa 153, 90146 Palermo, Italy; 2 Dipartimento di Scienze e Tecnologie Biologiche Chimiche e Farmaceutiche (STEBICEF), Università Degli Studi di Palermo, Viale Delle Scienze, 90128 Palermo, Italy; 3 Dipartimento di Biomedicina, Neuroscienze, e Diagnostica Avanzata (Bi.N.D) (sez. Anatomia Umana), Università di Palermo, via del Vespro 129, 90127 Palermo, Italy; 4 Centro di Ricerche Nutriterapiche, 61029 Urbino, Italy

**Keywords:** High-Fat Diet, neurodegeneration, AFA extract, supplementation, inflammation, astrocytes, microglia, amyloid beta

## Abstract

Obesity and related metabolic dysfunctions are associated with neurodegenerative diseases, such as Alzheimer’s disease. *Aphanizomenon flos-aquae* (AFA) is a cyanobacterium considered a suitable supplement for its nutritional profile and beneficial properties. The potential neuroprotective effect of an AFA extract, commercialized as KlamExtra^®^, including the two AFA extracts Klamin^®^ and AphaMax^®^, in High-Fat Diet (HFD)-fed mice was explored. Three groups of mice were provided with a standard diet (Lean), HFD or HFD supplemented with AFA extract (HFD + AFA) for 28 weeks. Metabolic parameters, brain insulin resistance, expression of apoptosis biomarkers, modulation of astrocytes and microglia activation markers, and Aβ deposition were analyzed and compared in the brains of different groups. AFA extract treatment attenuated HFD-induced neurodegeneration by reducing insulin resistance and loss of neurons. AFA supplementation improved the expression of synaptic proteins and reduced the HFD-induced astrocytes and microglia activation, and Aβ plaques accumulation. Together, these outcomes indicate that regular intake of AFA extract could benefit the metabolic and neuronal dysfunction caused by HFD, decreasing neuroinflammation and promoting Aβ plaques clearance.

## 1. Introduction 

The increase in lifespan leads to a growth in the incidence of age-related diseases, including neurodegenerative diseases. These disorders significantly impact the quality of life of patients and their caregivers and relatives, becoming a social and economic burden. However, aging is not the only risk factor for neurodegenerative diseases onset. Increasing evidence in humans and animals has shown a robust correlation between obesity and the development of neurodegenerative diseases, such as Alzheimer’s disease (AD) [1,2]. This pathology is characterized by progressive cognitive and memory loss that ultimately ends in dementia [3]. At the root of this condition is the widespread loss of neurons and their synapses in the particular brain area known as the hippocampus and entorhinal cortex. AD histopathological hallmarks are the so-called senile plaques, and neurofibrillary tangles obtained, respectively, by deposition of the aggregated β amyloid peptide (Aβ) and hyperphosphorylated Tau protein [4].

Metabolic changes caused by overweight and unhealthy lifestyle habits are associated with central nervous system (CNS) dysfunction, leading to neuronal death and alteration of synaptic plasticity that impairs memory ability. Intake of foods rich in fats and sugars and poor in vitamins and minerals are major risk factors for obesity and associated neurodegeneration [5,6]. 

The standard biological and molecular mechanisms involved in obesity and neurodegenerative diseases include insulin resistance, inflammatory cytokines activation, oxidative stress generation, mitochondrial dysfunction, and cell death. Brain insulin resistance is when brain cells fail to respond to insulin [5,7]. In AD and related disorders, insulin resistance is due to impaired insulin signaling, so AD is denominated as brain diabetes or “Type 3 Diabetes” [8,9]. In addition, central insulin resistance induces mitochondrial alterations, such as mitophagy, mitochondrial quality control, mitochondrial dysfunction, and apoptosis [10]. In agreement with these outcomes, High-Fat Diet (HFD) consumption causes cognitive impairments, reduces synaptic plasticity, induces neuroinflammation, and changes mitochondrial function and astrocytes activation [11,12]. However, the involved mechanisms are not well clarified, and several studies seek discoveries regarding their pathophysiology and prevention.

Since an unhealthy diet is the cause of metabolic dysfunctions and the development and progression of associated comorbidity, nutrition rich in antioxidant and anti-inflammatory compounds could help to reduce the risk of metabolic diseases, such as obesity or Type 2 Diabetes (T2D), and protect from related neurodegenerative disorders. The beneficial effect of antioxidant phytochemicals, as components of functional foods or used in supplementation, on obesity, neurodegeneration, and related comorbidity has been demonstrated in in vitro and in vivo model systems [13,14,15,16,17,18]. 

Recent data showed that the daily consumption of pistachios and honey could increase obesity-related dysmetabolic conditions, such as T2D, adiposity, and neurodegeneration in an animal model of diet-induced obesity [19,20,21,22,23,24,25,26].

Among supplements with potent antioxidant and anti-inflammatory effects, the extracts of blue-green algae play a relevant role. Spirulina, for example, contains numerous bioactive molecules, including beta-carotene, phycocyanin, tocopherols, micronutrients, fatty acids, and phenolic compounds [27]. It possesses antioxidant and anti-inflammatory properties and lipid-lowering ability. Further, its benefits on obesity and neurodegeneration can be extended to antiviral, anticancer, antidiabetic, hepatoprotective, and cardioprotective properties [27].

Recently, a growing interest has existed in the *Aphanizomenon flos-aquae* (AFA), another blue-green alga. AFA is a cyanobacterial unicellular organism endowed with several health-enhancing properties and spontaneously grows in Upper Klamath Lake (southern Oregon, USA). AFA contains all the vitamins; it is the only living food with 72 minerals; and it has the most comprehensive spectrum of carotenes, such as beta-carotene, xanthophyll, and an unusually high concentration of chlorophyll [28,29]. Among its bioactive molecules, particularly relevant are phenylethylamine, an important neuromodulator, and a particular type of AFA phycocyanin, also composed of phycoerythrocyanin, with very high antioxidant, anti-inflammatory and antiproliferative properties, that are reinforced by the further presence of mycosporine-like amino acids (MAAs) and various polyphenols [30,31,32,33].

Further, it has been reported that the AFA extract Klamin^®^ which concentrates phenylethylamine, can influence mood, reduce anxiety, and enhance attention and learning, suggesting that it could have a role in clinical brain areas [34,35]. An in vitro study on neuronal cells stimulated with Aβ demonstrated that the AFA extract Klamin^®^ plays a protective role in neurodegenerative processes, such as oxidative stress generation, inflammation, and formation of amyloid plaques [36]. 

Further, the possibility of using the AFA extract to develop functional food for the health and wellness market has been evaluated. Another study considered employing the AFA extract as an additive in biscuit dough, demonstrating that AFA antioxidant properties are also maintained after exposure to high temperatures [37]. 

Moreover, cellular molecules and mechanism that joins dysmetabolism and obesity-related neurodegeneration have yet to be thoroughly explored. Neuroinflammation is closely related to the pathogenesis of AD and obesity/T2D [38]. In the brain, astrocytes and microglia cells maintain homeostasis and support many functions of neurons. Moreover, they play an essential role in the inflammatory process of neurodegenerative diseases [39]. 

Recently a new AFA product reached the market, KlamExtra^®^, which combines the Klamin^®^ extract (EU patent n° 2046354), which has more specific neuromodulatory and immunomodulatory properties, with the AphaMax^®^ extract (EU patent n° 2032122), which concentrates the AFA-phycocyanins and, so, has increased antioxidant and anti-inflammatory properties.

Here, we aimed to study the effect of KlamExtra^®^ on the molecular mechanisms involved in neurodegeneration induced by obesity. Further, by using glial fibrillary acid protein (GFAP) and soluble triggering receptors expressed on myeloid cells-2 (sTREM-2) as biomarkers, we explored the possibility that AFA can mitigate the central inflammatory process induced by the HFD diet by modulating astrocytes and microglia activation. A possible protective response of AFA in reducing Aβ deposits was also explored. From now on, we shall define KlamExtra^®^ as AFA.

## 2. Results 

### 2.1. AFA and Metabolic Parameters 

**Figure 1:**
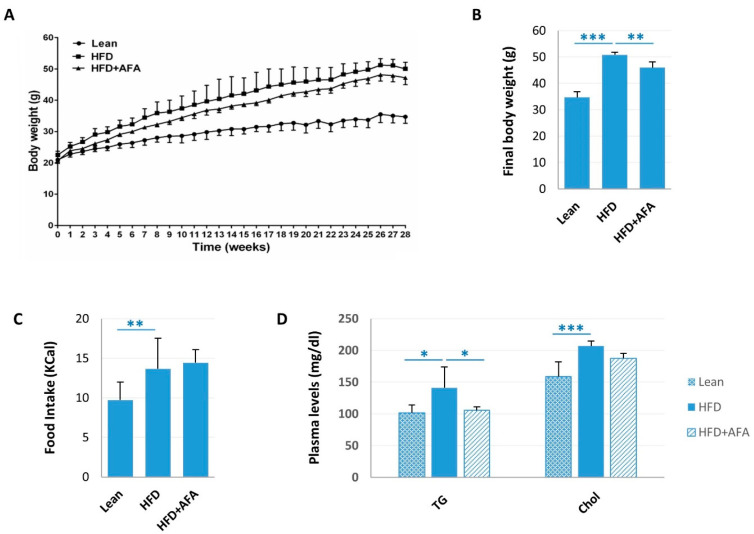
Effects of AFA chronic ingestion on body weight, food intake, and plasma lipids in HFD mice. (**A**) Body weight changes. (**B**) Final body weight. (**C**) Food intake. (**D**) Triglyceride (TG) and total cholesterol plasma (Chol) levels. Data are mean values ± S.E.M. (*n* = 8 mice/group). * *p <* 0.05, ** *p <* 0.01, *** *p <* 0.001 vs. STD mice and vs. HFD-fed mice.

The effects of the AFA assumption on animal body weight, food intake, and circulating lipids are illustrated in Figure 1. HFD animals gradually and more rapidly enhanced throughout the twenty-eighth week compared with the Lean group. In AFA-fed mice, the body weight gain was lower than in HFD mice (Figure 1A). At the end of the experimental protocol, the body weight mean values were 34.74 ± 2.14 g for Lean mice, 50.85 ± 1.00 g for HFD animals, and 46.00 ± 2.20 g for the HFD supplemented with AFA groups (Figure 1B). Food intake was approximately similar between the HFD and HFD + AFA groups, but it was significantly different from the Lean group (Figure 1C). Plasma Triglycerides (TG) and Cholesterol (Chol) levels were higher in HFD mice compared with the Lean group or the HFD + AFA group (Figure 1D). 

**Figure 2:**
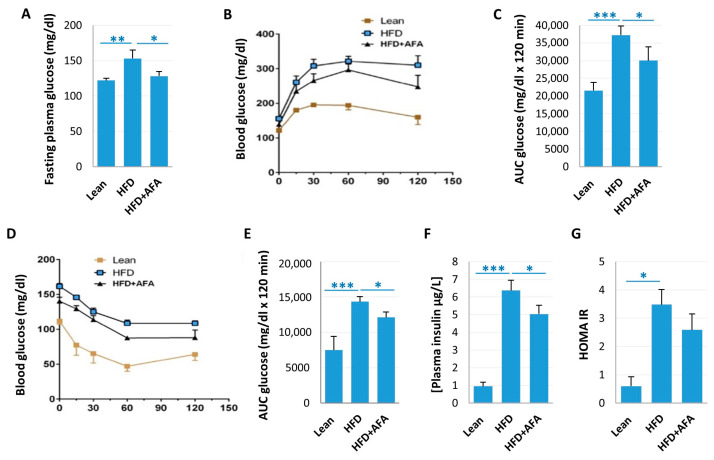
AFA assumption improves glucose dysmetabolism in HFD mice. Fasting glycaemia (**A**), glucose tolerance test (GTT) (**B**), area under the curve (AUC) for GTT (**C**), insulin tolerance test (ITT) (**D**), area under the curve for ITT (**E**), plasma insulin levels (**F**) and HOMA index (**G**). Results are shown as means ± SEM of 8 animals/group. * *p <* 0.05, ** *p <* 0.01, *** *p <* 0.001 vs. STD mice and vs. HFD-fed mice.

To investigate the effects of AFA on glucose homeostasis, we measured the fasting plasma glucose and performed intraperitoneal glucose and insulin tolerance tests. Interestingly, in the AFA-supplemented HFD group, fasting blood glucose concentration (128.3 ± 6.23 mg/dL) was lower than in the HFD group (153 ± 11.78 mg/dL), and there was no statistically significant difference between Lean animals (122 ± 3 mg/dL) and the HFD + AFA group (Figure 2A).

Figure 2B represents the blood glucose concentrations over 2h after i.p. glucose injection in different mice. The glucose tolerance curve and the related AUC in HFD were significantly higher compared with Lean mice (Figure 2B,C), indicating an impairment of glucose tolerance. The curve and AUC in HFD + AFA were considerably lower than in HFD, suggesting a beneficial effect on glucose homeostasis.

During the insulin tolerance test, HFD mice displayed higher blood glucose concentration than Lean mice (Figure 2D,E), suggesting impaired insulin sensitivity. In HFD + AFA mice, we found lower glycemic values and decreased AUC after insulin injection, suggesting an improved insulin sensitivity (Figure 2D,E).

Insulin concentrations were significantly higher in HFD in comparison with Lean and HFD + AFA mice (Figure 2F). In addition, HOMA-IR was slightly ameliorated in HFD + AFA mice (Figure 2G). These results indicate that AFA chronic ingestion improves insulin resistance and glucose intolerance in HFD mice.

### 2.2. AFA Improves Brain Insulin Resistance in HFD Mice

**Figure 3:**
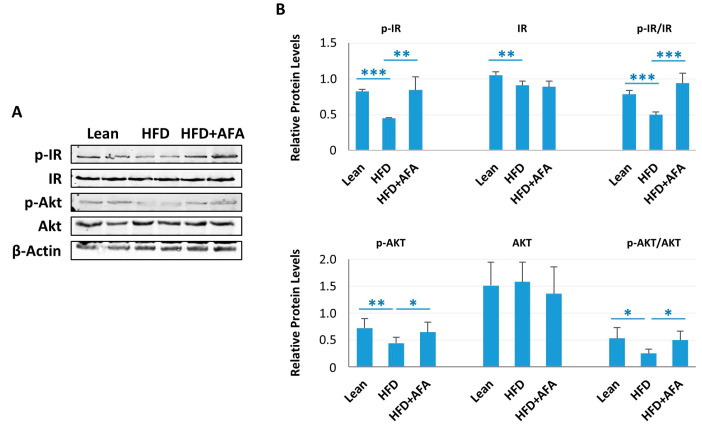
AFA chronic ingestion reduces insulin resistance. (**A**) Immunoblot of total lysate of Lean, HFD and HFD + AFA cortex incubated with anti-IR, anti-phospho-IR (p-IR), anti-Akt, anti-phospho-Akt (p-Akt) and anti-β-actin. (**B**) Quantification of immunoreactivity was performed by using densitometric analysis; uniformity of gel loading was confirmed with β-actin utilized as standard. * *p* < 0.05, ** *p* < 0.005, *** *p* < 0.001 vs. indicated groups.

A reduction of insulin receptor expression and impairment of insulin signaling characterizes insulin resistance. In the brain, insulin resistance has been associated with neurodegenerative disorders [2]. In HFD-fed mice, phosphorylated brain insulin receptor (p-IR) protein expression was decreased compared with the control group, suggesting that cerebral insulin resistance is diet-induced. In contrast, the HFD + AFA group showed a level of expression of p-IR similar to the Lean group (Figure 3A,B).

Furthermore, we analyzed the expression of proteins involved in insulin signaling in the brain. Decreased levels of posho-Akt were found in HFD mice compared with the Lean group (Figure 3A,B). In contrast, HFD + AFA-fed mice showed higher levels of phospho-AKT, suggesting that AFA extract ingestion can counteract HFD-induced brain insulin resistance (Figure 3A,B).

### 2.3. AFA Consumption Induces Neuroprotection

**Figure 4:**
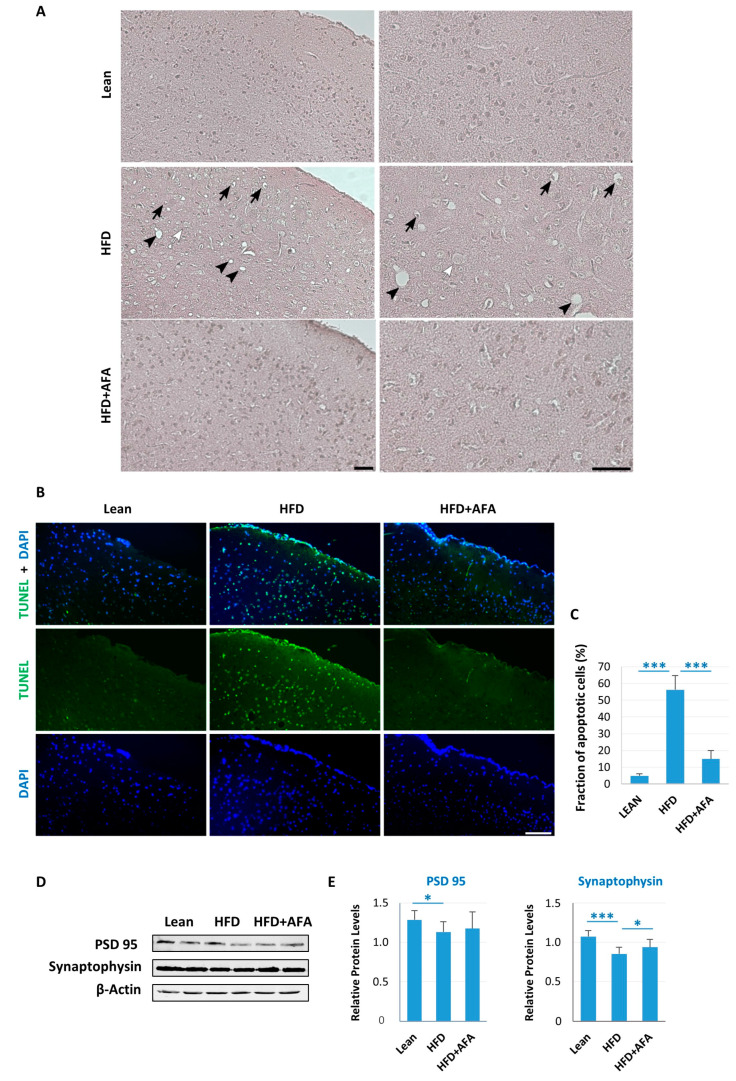
AFA ameliorates degeneration in the brain of diet-induced obese mice. (**A**) Morphology assessments on the cortex of Lean, HFD and HFD + AFA mice with hematoxylin and eosin stain. In the HFD group, there are numerous pycnotic (black arrows) and swollen (white arrows) cells, while strong vacuolation (arrowheads) was observed in all cortical layers. A sharp reduction in degenerative signs is observed in the sections of the AFA-treated group; (**B**) TUNEL assay on cerebral cortex sections; (**C**) Number of apoptotic nuclei in the cerebral cortex of Lean, HFD and HFD + AFA mice. Data are mean values ± S.E.M. (n = 6/group). *** *p <* 0.001. Scale bar, 50 μm; (**D**) Western blot of protein extracted from brain lysates of different mouse groups and incubated with anti-PSD95, anti-synaptophysin, and anti-*β*-actin antibodies; and (**E**) Quantification of immunoblot was performed by using densitometric analysis; uniformity of gel loading was confirmed with β-actin utilized as standard. Results are shown as means ± SEM of 6 animals/group. * *p* < 0.05, *** *p* < 0.001 vs. indicated groups.

The effect of the different diets on brain morphology was analyzed by staining with Hematoxylin–Eosin. Histopathological analysis showed, in the cortex of the HFD group, damaged/disorganized neurons and the presence of numerous pyknotic cells. Further, a robust vacuolization in other brain cortical layers was observed. In contrast, in the HFD + AFA group, neuronal morphology was comparable to the Lean controls, except for certain pyknotic cells (Figure 4A). Further, a clear-cut result regarding the protective role of AFA extract on neurons was obtained by TUNEL assay. A significantly increased number of fragmented nuclei were detected in the cerebral cortex of the HFD group compared with the Lean and HFD + AFA mice, suggesting that AFA extract consumption can attenuate the degenerative neuronal process induced by the HFD diet (Figure 4B,C).

Furthermore, synaptic loss is present in obesity-related neurodegeneration [40]. The presynaptic protein synaptophysin and the postsynaptic protein PSD95 were downregulated in the HFD-fed mice compared with the Lean group. In contrast, HFD + AFA-fed mice exhibited a significant increase in synaptophysin. Although it does not reach significance, a trend towards an increase in PSD95 levels has been observed, suggesting a beneficial effect of AFA extract on synaptic transmission (Figure 4D,E). 

### 2.4. AFA Reduces Aβ Accumulation

**Figure 5:**
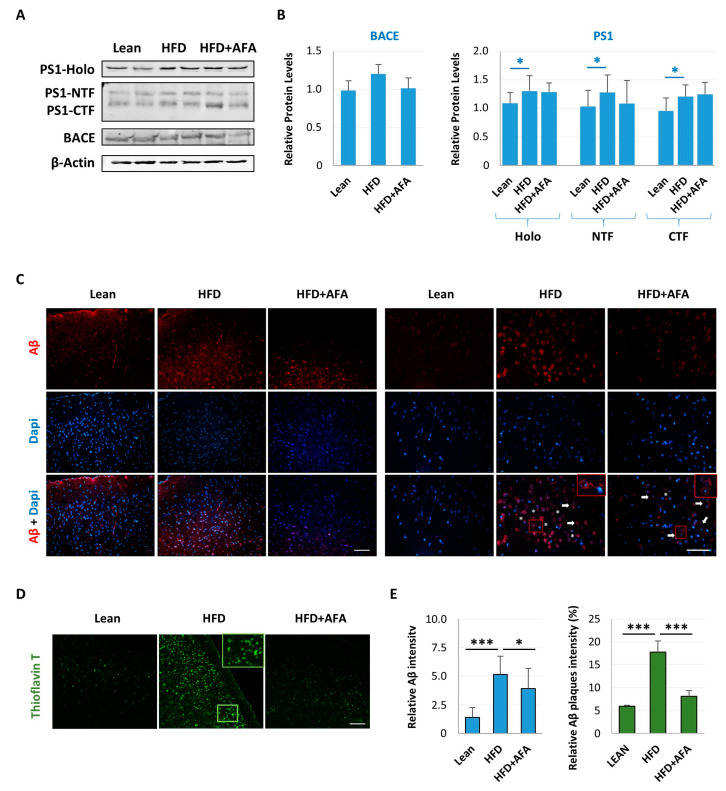
AFA treatment reduces Aβ deposition in HFD mouse brains. (**A**) Immunoblot of total lysate of Lean, HFD and HFD + AFA cortex incubated with anti-PS1, anti-BACE1 and anti-β-actin; (**B**) Quantification of immunoreactivity was performed using densitometric analysis; uniformity of gel loading was confirmed with β-actin utilized as standard. * *p <* 0.05, for Holo protein and PS1 fragments HFD vs. Lean; (**C**) Representative fluorescence images of the cortex of Lean, HFD and HFD + AFA mice. The Aβ fluorescence signal appears in the intracellular compartment of the cortex cells (arrows) in HFD + AFA mice and extracellular Aβ deposits are also present (asterisks) in HFD mice. Nuclei were stained with DAPI. Scale bars, 50 μm and 20 μm. (**D**) Thioflavin T staining of Aβ aggregates on cerebral cortex section of Lean, HFD and HFD + AFA mice. The outlined area in D is enlarged to the right to show the stained plaque. Thioflavin T-positive amyloid deposits are prominent in cortex areas of HFD mouse compared with those from Lean and HFD + AFA counterparts. Scale bar, 100 μm. The outlined areas in C and D are enlarged. (**E**) The number and intensity of Aβ plaques detected by Thioflavin T were quantified using Image J software. All images were taken using a fluorescence microscope. Asterisks indicate significant differences between HFD vs. Lean and HFD + AFA vs. HFD (* *p* < 0.05, *** *p* < 0.001).

We also examined the levels of expression of BACE1 and PSN1, two enzymes involved in processing APP and Aβ production [41]. Although not significant, BACE1 expression shows an upward trend in the HFD compared with the HFD + AFA group (Figure 5A,B). Furthermore, in the HFD brain, PS1 expression levels showed an increase in both the whole protein (Holo) and the proteolytic fragments NTF and CTF. In the HFD + AFA group, the NTF fragment was reduced (Figure 5A,B). 

Further, we investigated neuronal APP-Aβ presence in the brain of different animal groups. Aβ immunoreactivity was reduced in the Lean and HFD + AFA groups compared with the HFD mice. In addition, in the Lean and HFD + AFA groups, we observed diffuse staining around the nuclei, indicating accumulation of intraneural Aβ. In contrast, in HFD mice, an APP punctate staining around the neurons was found, suggesting an increase in APP processing and Aβ aggregation (Figure 5C). Furthermore, this result was validated by staining with Thioflavin T (Figure 5D), a dye used to visualize the presence of β-sheet protein aggregates or amyloid plaques, whose existence was detected mainly in HFD mice.

### 2.5. AFA Counteracts Neuroinflammation

**Figure 6:**
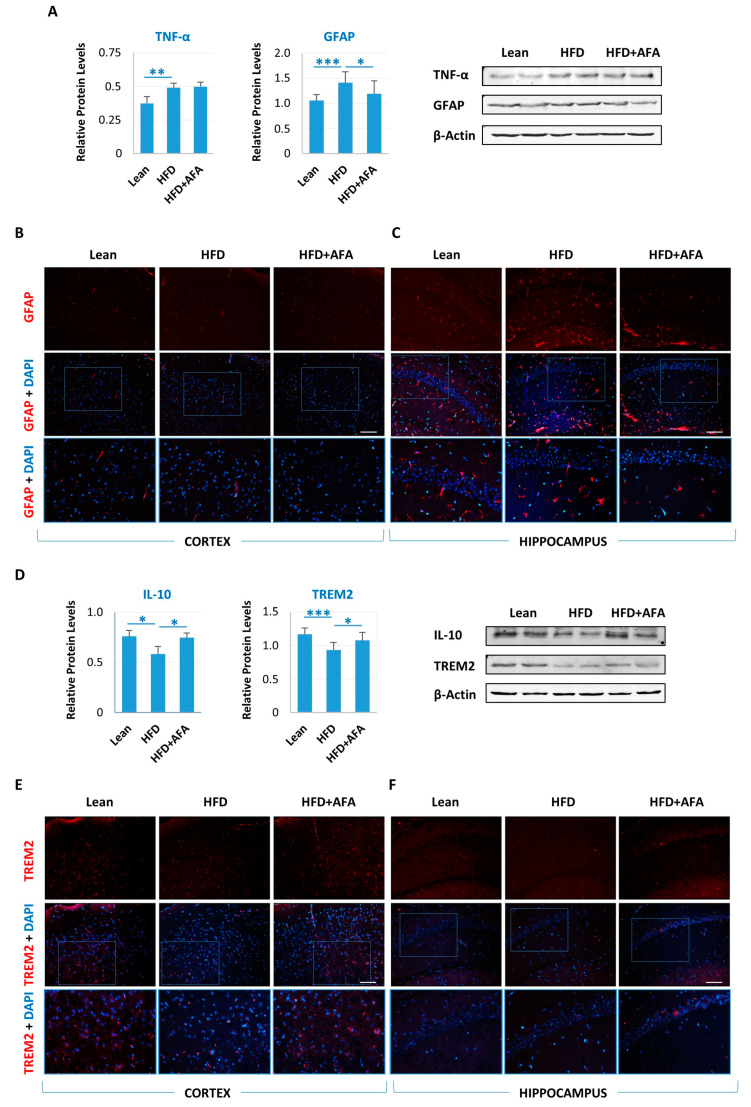
AFA improves inflammation induced by HFD in mouse brains. (**A**) Immunoblot of total lysate of Lean, HFD, and HFD + AFA cortex incubated with anti-TNFα, anti-GFAP, and anti-β-actin. Quantification of immunoreactivity was performed by using densitometric analysis; uniformity of gel loading was confirmed with β-actin utilized as standard. * *p* < 0.05, ** *p* < 0.005, *** *p <* 0.001 HFD vs. Lean and HFD + AFA vs. HFD; Representative GFAP fluorescence images of the cortex (**B**) and hippocampus (**C**) of Lean, HFD, and HFD + AFA mice. (**D**) Immunoblot of total lysate of Lean, HFD, and HFD + AFA cortex incubated with anti-IL-10, anti-TREM2, and anti-β-actin. Quantification of immunoreactivity was performed by using densitometric analysis; uniformity of gel loading was confirmed with β-actin utilized as standard. * *p* < 0.05, *** *p* < 0.001 HFD vs. Lean and HFD + AFA vs. HFD; Representative TREM2 fluorescence images of the cortex (**E**) and hippocampus (**F**) of Lean, HFD, and HFD + AFA mice. Nuclei were stained with DAPI. Scale bars, 50 μm. The outlined areas in (**B**,**C**,**E**,**F**) are enlarged to show astrocytes and microglia.

Peripheral inflammation triggered by obesity is associated with neuroinflammation [5]. Increased expression of TNF-α (Figure 6A) and decreased expression of IL-10 (Figure 6D) were detected in the brain of HFD-fed mice as compared with the Lean group, indicating activation of the inflammatory response. In contrast, in the HFD + AFA group, an expression level similar to that of the Lean mice was found for IL-10 (Figure 6D).

Further, we analyzed the expression of GFAP, a biomarker for the activation of astrocytes [42]. The increase of GFAP expression observed in the HFD group with respect to the lean group was significantly counteracted by AFA consumption (Figure 6A). Accordingly, immunofluorescence analysis showed an increase in GFAP intensity mainly in the hippocampus (Figure 6C) of HFD-fed mice compared with the Lean group. In addition, a significant reduction of fluorescent intensity in the brain of HFD + AFA-fed mice indicate that the increase of astrocytes in response to HFD can be counteracted by adding an AFA supplement to the food (Figure 6A–C).

Further, we analyzed the expression levels of TREM2, a receptor expressed mainly on microglia and modulated in obesity-induced insulin resistance [43], a condition in which TREM2 exerts anti-inflammatory and neuroprotective effects. Consistent with the IL-10 result, TREM2 decreased in the brains of the HFD group. In contrast, higher levels of this protein were detected in HFD + AFA-fed mice brains (Figure 6D). We also observed higher TREM2 immunoreactivity especially in the cortex of the Lean and HFD + AFA mice than the HFD group, suggesting a protective effect on microglial cell viability (Figure 6E). All these results indicate that the bioactive molecules contained in AFA extract can have an impact on multiple mechanisms of the neuro-inflammation process and promote the healthy neuronal homeostasis.

### 2.6. AFA Modulates Astrocytes and Microglia Activation and Aβ Deposition

**Figure 7:**
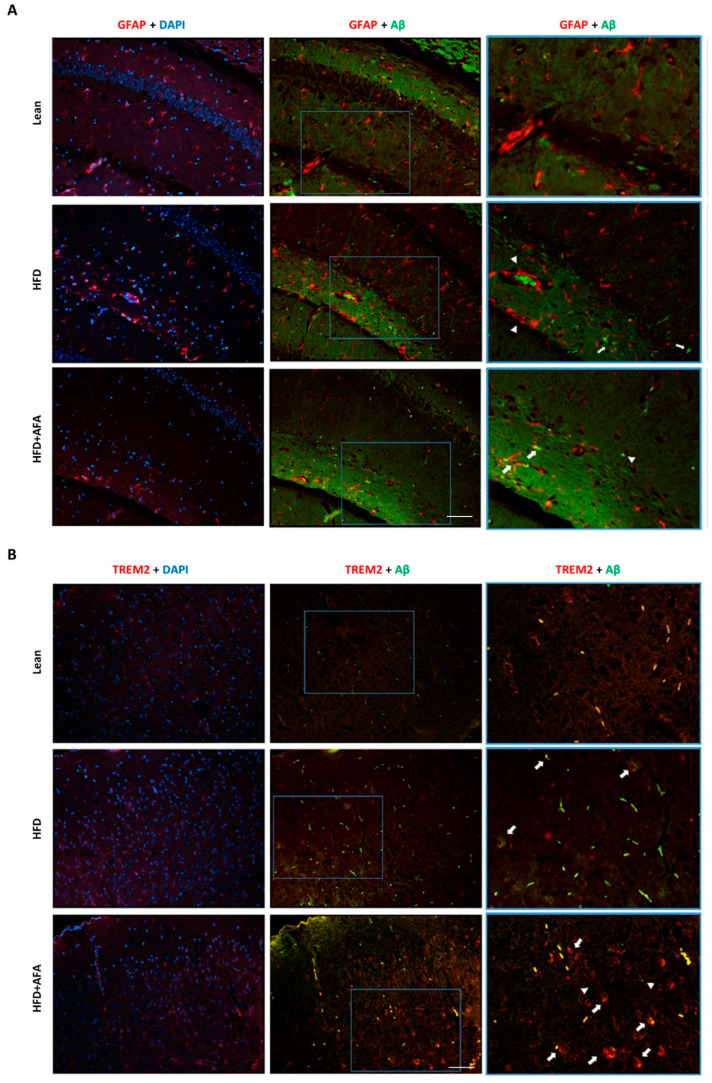
AFA modulates astrocytes and microglia activation induced by HFD in mouse brains. Representative fluorescence images of GFAP + Aβ (**A**) in the hippocampus and TREM2 + Aβ (**B**) in the cortex of Lean, HFD, and HFD + AFA mice. The outlined areas in A and B are enlarged to show the co-staining. Arrows indicate colocalization of astrocytes and microglia with Aβ plaques. Arrowheads indicate Aβ plaques not associated with astrocytes. Nuclei were stained with DAPI. Scale bars, 50 μm.

Hippocampus brain sections of Lean, HFD, and HFD + AFA-fed mice were used for GFAP and Aβ staining. Analysis of GFAP immunoreactivity astrocytes increase in response to the HFD diet and the presence of Aβ deposition that was attenuated in HFD + AFA-fed mice (Figure 7A,B). We also examined the presence of TREM2 and Aβ accumulation in the cortex of the different groups. TREM2 reduction observed in HFD-fed mice affected the clustering of microglia around the Aβ deposits, which was improved by AFA supplementation (Figure 7C,D).

## 3. Discussion

Growing evidence indicates how unhealthy nutrition can be considered a potential cause of metabolic-related disease and disorders of the central nervous system (CNS). A correct lifestyle and constant physical exercise can prevent these pathologies, and nutritional supplements can help with this. Here, we explored the impact of the two AFA extracts (KlamExtra^®^) intake in the brain of obese mice. We applied an HFD feeding protocol to induce obesity and neurodegeneration in mice. We observed the preventive action of AFA extract as a dietary supplement in reducing brain metabolic and molecular impairment. AFA bioactive molecules have the chemical characteristics to cross the BBB, and its dietary consumption can be seen as a way to compensate for the loss of efficacy of the endogenous defenses [44]. 

A slight but significant reduction in body weight after regular food intake of KlamExtra^®^ was observed in comparison with the obese control group. A considerable reduction of plasma triglyceride levels was also observed in the obese group supplemented with AFA. A high amount of dietary fiber and anti-obesity active compounds, including carotenoids, could account for the positive effect of these algae. Analysis of metabolic parameters showed that HFD fasting glycemia, insulin concentration, and HOMA index were more elevated than in Lean mice, indicating an impairment of glucose metabolism and insulin resistance condition, which was lightly improved by AFA consumption. Thus, the effects of regular AFA extract intake can be assigned to beneficial actions on glucose metabolism.

In line with previous results and in accordance with metabolic data, we demonstrated that brain dysfunction in long-term HFD-fed mice is associated with peripheral and central insulin resistance [2]. Brain insulin resistance in HFD-fed mice at molecular levels was confirmed by the reduced expression of insulin receptors and molecules involved in insulin signaling, such as Akt/p-Akt. In contrast, a supplement of AFA extract counteracted insulin sensitivity and insulin signaling impairment.

It has been widely reported that HFD consumption causes increased neuroinflammation and neuron loss [45]. AFA seems to reduce the neuroinflammatory profile modulating the expression of cytokines and activating astrocytes and microglia through the action of GFAP and TREM2 proteins. An increase of TREM2 and a decrease of GFAP expression in the AFA group suggest a protective reaction to the damage of brain homeostasis induced in the HFD model. In addition, our results could indicate that AFA protects from Aβ injury produced by the HFD diet by promoting microglial clearance. 

AFA consumption mitigated degeneration and loss of neurons induced by the HFD diet, as demonstrated by histopathological analysis and TUNEL assay. Further, in the HFD group, loss of neurons was associated with loss of synapses, as suggested by the reduced expression of PSD95 and synaptophysin that was prevented by AFA addition, signifying a beneficial effect of the supplement on neurons’ health and communication. 

Consistent with this result, we found that specific proteins related to APP processing, including BACE1 and PSN1, were up-regulated in the brain of HFD-fed mice. In contrast, although not significant, their expression level was reduced in HFD + AFA mice. The increased presence of these proteins is associated mainly with the augmented production of Aβ. This complies with the accumulation of extracellular insoluble Aβ fibrillar aggregates and amyloid plaques found in the cerebral cortex of HFD brain sections and evidenced by ThT staining. In contrast, Aβ intracellular presence in the HFD + AFA group suggested that AFA could exert a neuroprotective role by interfering with APP processing and Aβ aggregation.

GFAP is an astrocyte protein overexpressed after a neurological insult known as astrogliosis [46,47]. The astroglial reaction has been observed in a different case in which memory performance is weakened [48]. We found an increase of GFAP and astrocytes in response to hypercaloric feeding suggesting a neuroinflammatory state and the occurrence of astrogliosis that was prevented by AFA supplementation. It has been reported that unhealthy nutrients and metabolites in an HFD diet can impact brain function by crossing the blood–brain barrier (BBB), interacting with neurons and triggering glia [49]. AFA extract intake could reduce or eliminate metabolites peripherally generated by the HFD diet which, no longer being transported to the brain, elude astrogliosis. In addition, our results agree with the finding that growth factors and cytokines, such as IL-1, IL-6, TNF-α, and reactive oxygen species are among the main signaling molecules that can regulate astrogliosis [46,47]. 

Several studies have demonstrated that TREM2 protects against neurodegeneration by controlling neuroinflammation closely related to the pathogenesis of AD and obesity [50]. 

Overexpression of TREM2 was reported to upregulate synaptic proteins synaptophysin and PSD95, improving synaptic transmission in long-term HFD-fed mice, whose loss is related to memory and learning impairment [51]. Similarly, supplementation of AFA to HFD upregulates TREM2 and synaptic proteins, suggesting that it supports neuron metabolism and synapses. 

While astrocytes have been assigned the role of filling tissue voids caused by degenerative events, microglia, the brain’s immune cells, function as brain phagocytes responsible for removing debris from degenerating neurons and Aβ deposit that interferes with neuron communication [52]. 

Further, it was also demonstrated that TREM2 is essential for promoting microglial clustering around fibrillar Aβ plaques in AD mouse models and postmortem human brain sections [53]. Its deletion induces a reduction in plaque-associated microglia [53,54]. In addition, during AD development, homeostatic microglia respond to Aβ accumulation evolving into disease-associated microglia (DAM) [55]. 

This is consistent with the immunofluorescence results in which, in the AFA group, an increase of TREM2 is associated with a reduction of the formation of Aβ deposits. Further, the data examined here indicate that TREM-2 regulates microglia activation in response to dietary factors.

Recently, a study on patients affected by neurogenerative diseases has evidenced altered levels of GFAP and TREM-2 in CSF, suggesting that they could be used as biomarkers of central inflammation and have been proposed as prognostic tools of neurodegenerative progression [56]. 

However, we cannot exclude that the improved metabolic conditions and the restored homeostasis observed in the brain of the HFD + AFA group might have slowed down the APP processing and Aβ deposition, favoring the microglia response. 

In conclusion, our results suggest that KlamExtra^®^, a natural product, works as a “functional food” to activate a compensatory mechanism mainly for mitigating HFD-induced systemic and central dysmetabolism.

AFA extract can alleviate central neuroinflammation HFD- induced by regulating astroglial and microglial activation and modulating anti-inflammatory cytokines. These outcomes associated with increased synaptic protein expression and Aβ plaques removal suggest that AFA extract could have promising protective activity on neurodegenerative diseases. However, additional investigations are necessary to confirm our findings. 

## 4. Materials and Methods

### 4.1. Animals and Diets 

All animals received care in compliance with the recommendations of the European Economic Community (2010/63/UE) and the guidelines for animal experimentation (Italian D.L. No. 26/2014 and subsequent variations). The Ministry of Health authorized the experimental protocol (Rome, Italy; Authorization Number 46/2020-PR, date of approval: 21 January 2020).

KlamExtra^®^ includes the two AFA extracts Klamin^®^ and AphaMax^®^ whose composition was previously described in EU patents n° 2046354and 2032122 and by Nuzzo et al. [36].

Four-weeks-old male C57BL/6J mice were purchased from Harlan Laboratories (San Pietro al Natisone, Udine, Italy). As described in previous papers [22,23], after a 1-week habituation period, the animals were weighed and divided into separated three groups: (A) Lean group (Lean, *n* = 8) fed a standard diet consisting of 70% of energy as carbohydrate, 20% proteins and 10% fat (code 4RF25, Mucedola, Milan, Italy); (B) High-Fat Diet group (HFD, *n* = 8) fed HFD (PF4215, Mucedola, Milan, Italy) that supplied 60% of energy as fat, 20% proteins and 20% carbohydrates; (C) mice fed a HFD supplemented with KlamExtra^®^ (HFD-AFA, *n* = 8) for 28 weeks. HFD-AFA was custom designed and prepared by Mucedola S.r.l (4RF25), obtained by adding 8.3 g of KlamExtra/Kg HFD. All animals (two animals per cage) were maintained under a 12 h dark–light cycle at 23 ± 1 °C and 55% ± 5% humidity, with free access to food and water ad libitum. 

Bodyweight and food intake were detected weekly throughout the study. At the end of the study period, metabolic parameters were analyzed, then the animals were sacrificed by cervical dislocation. Blood was immediately drawn by cardiac puncture, and plasma was recovered after centrifugation at 3000 rpm at 4 °C for 15 min and stored at −80 °C until analysis. The aorta was cannulated and perfused with Dulbecco’s buffered solution containing 2 mM EDTA, and the right atrial incision allowed blood outflow. Brains were explanted, weighed, and processed for subsequent analysis. AFA (0.9 mg/mouse) was administered daily for 28 weeks. The doses given to the Diet-Induced Obese (DIO) mice were extrapolated from the human dosage (1.6 g/day) and calculated on the basis of the average body weight (40 mg) [22].

### 4.2. Metabolic Parameters

Plasma triglyceride and total cholesterol were measured using the ILAB 600 Analyzer (Instrumentation Laboratory, Bedford, MA, USA). Fasting blood glucose concentrations were determined by a glucometer (GlucoMen LX meter, Menarini, Florence, Italy). Intraperitoneal glucose tolerance test (IPGTT) and insulin tolerance test (ITT) were carried out in overnight-fasting mice. For IPGTT, mice were injected intraperitoneally (i.p.) with glucose (2 g/kg b.w.) (D-glucose, Sigma-Aldrich, Milan, Italy) in 0.9% saline. For ITT, mice were injected i.p. with insulin (0.5 U/kg b.w.) (Insuman Rapid, Sanofi Aventis, Italy) in 0.9% saline. Tail-vein-measured glucose concentrations were taken at different time points (0, 15, 30, 60, and 120 min). Plasma insulin was quantified using a mouse ELISA kit (Alpco diagnostics, Salem, NH, USA) according to the manufacturer’s instructions, and the HOMA-IR, index of insulin resistance, was calculated as the ratio of fasting insulin (ng/mL) and fasting glucose (mg/dL) divided by the constant 22.5. 

### 4.3. Total Protein Extraction and Western Blot

Brain tissue from Lean, HFD and HFD + AFA mice was homogenized in RIPA buffer (20 mM Tris-HCl pH 7.4, 150 mM NaCl, 1 mM Na3VO4, 10 mM NaF, 1mM EDTA, 1 mM EGTA, 0.2 mM phenylmethylsulfonyl fluoride, 1% Triton, 0.1% SDS, and 0.5% deoxycholate) with protease inhibitor (Amersham, Life Science, Les Ulis, France) and phosphatase inhibitor cocktail (Sigma-Aldrich, Poole, Dorset, UK). The samples were sonicated and centrifuged for 30 min at 14,000 rpm at 4 °C to remove insoluble material, and the supernatants were quantified by the Bradford method (Bio-Rad) and collected. Proteins (50 μg) were separated by 10% or 12% acrylamide gel and were transferred onto a nitrocellulose filter. The filter was incubated with the anti-insulin receptor (IR) (1:1000, Invitrogen, Waltham, MA, USA), anti-phospho insulin receptor (p-IR) (1:1000, Invitrogen, Waltham, MA, USA), anti-protein kinase B (Akt) (1:1000, Cell Signaling Technology, Danvers, MA, USA), anti-phospho protein kinase B (p-Akt) (1:1000, Cell Signaling Technology, Danvers, MA, USA), anti-presenilin1 (PSN1) (1:200, Santa Cruz Biotechnology, Santa Cruz, CA, USA), anti-beta secretase enzyme 1 (BACE1) (1:500, Cell Signaling Technology, Danvers, MA, USA), anti-postsynaptic density protein 95 (PSD-95) (1:1000, Santa Cruz Biotechnology, Santa Cruz, CA, USA), anti-synaptophysin (1:1000, Santa Cruz Biotechnology, Santa Cruz, CA, USA), anti-tumor necrosis factor α (TNFα) (1:500, Thermo Fisher, Preprotech, Waltham, MA, USA), anti-glial fibrillary acidic protein (GFAP) (1:1000, Cell Signaling Technology Danvers, MA, USA), anti-interleukin 10 (IL-10) (1:500, Santa Cruz Biotechnology, Santa Cruz, CA, USA), an anti-triggering receptor expressed on myeloid cells 2 (TREM2) (1:1000, Invitrogen, Waltham, MA, USA), and anti-*β*-actin (*β*-Actin; 1:10,000, Sigma-Aldrich, St. Luois, MO, USA). Primary antibodies were detected using the Odyssey^®^ scanner (Li-cor), according to the manufacturer’s instructions, using secondary antibodies (anti-mouse and anti-rabbit) labeled with IR790 and IR680 (1:10,000; Life Technology, Carlsbad, CA, USA). Band intensities were analyzed with ImageJ software, and expression was adjusted to β-Actin expression. The protein levels were expressed as intensity relative to the control.

### 4.4. Histopathology and Immunohistochemistry

For histopathological and immunohistochemical analyses, whole-brain specimens/samples were collected and fixed in 4% paraformaldehyde in 0.1 M PBS (pH 7.4) overnight at 4 °C and then transferred into dehydrated in graded ethanol and paraffin-embedded. Serial sagittal sections (5μ thick) were deparaffinized in xylene, rehydrated with a graded series of ethanol, and then processed for routine hematoxylin and eosin (HE) used according to the manufacturer’s instructions. For immunofluorescence assays, sections were treated in 10 mM citrate buffer (pH 6.0) with microwaves for 6 min at 500 W for antigen retrieval. Then, cells were blocked with 10% normal goat (NGS) serum in PBS for 1 h at room temperature (RT) and were incubated at 4 °C overnight with anti-GFAP (1:200), anti-TREM2 (1:200), and anti-Aβ (1:100) primary antibodies (3% NGS in PBS and 0,3% Triton X-100, PBS-T). Immunopositive reactions were detected by incubation with the anti-rabbit Alexa 488 or anti-mouse Alexa 555 (1:500; Cell Signaling Technology) secondary antibodies (2% NGS in PBS) for 2 h at RT. 

Finally, all sections were coverslipped using Sigma mounting medium, including 4,6-diamidino-2-phenylindole (DAPI; Vector Laboratories), and visualized with Axioskop-2 Zeiss or Leica DM4000 microscope. All photomicrographs were collected using the same magnification (10×, 20×, or 40× objectives), exposure time, and other parameters, and the representative images from five sections for each brain specimen were edited with Adobe Photoshop software. Negative controls were performed for every set of experiments by omitting the primary antibodies.

For quantitative analysis, the images were imported into the ImageJ software program (NIH, Bethesda, MD), converted to grayscale, and the total area of immunoreactivity was quantified by measuring the mean intensity of all stained areas of each micrograph, and the data were expressed as mean density.

### 4.5. Thioflavin T Staining

Following deparaffinization with xylene and ethanol, tissue sections were incubated in 70 μM of Thioflavin T (ThT, Sigma-Aldrich, St. Louis, MO, USA) for 10 min at RT. Sections were then rinsed in 70% ethanol. After washing in deionized water, the slides were mounted in aqueous mounting media. The images were visualized by a Leica DM4000 microscope at 10× magnification. The number and area of plaques detected by ThT were quantified using Image J software.

### 4.6. TUNEL Assay

In situ detection of DNA fragmentation was performed using the DeadEnd™ Fluorometric TUNEL Detection System kit (Promega, Madison, WI, USA), following the manufacturer’s instructions. Pre-incubating sections performed positive controls with DNase I for 10 min at room temperature, and negative controls by omitting the TdT enzyme. Nuclei were stained with DAPI (Vector Lab, Burlingame, CA, USA), and the slides were analyzed using a fluorescent microscope (Leica Microsystems) at a magnification of 20X. TUNEL-positive cells were quantified and results were expressed as mean ± SEM values of three different experiments.

### 4.7. Statistical Analysis

The results are presented as the mean ± the standard error of the mean (SEM). The letter ‘n’ indicates the number of animals. Statistical analyses were performed using the Prism Version 6.0 Software (Graph Pad Software, Inc., San Diego, CA, USA). The comparison between the groups was performed by one-way Analysis of Variance (ANOVA), followed by Bonferroni’s post-test for significance analysis. Results with a *p*-value ≤ 0.05 were considered statistically significant. 

## Data Availability

The data presented in this study are available from the corresponding author on reasonable request.
